# Supplementary material for: Prolonged Length of Stay in the Emergency Department and Increased Risk of In-Hospital Cardiac Arrest: A nationwide Population-Based Study in South Korea, 2016–2017
Source: J Clin Med. 2020 Jul 18;9(7):2284. doi: 10.3390/jcm9072284 (PMC7408893; doi:10.3390/jcm9072284)
Supplement: Supplementary file 1 [file jcm-09-02284-s001.pdf]

Table S1. Baseline demographic and clinical characteristics of emergency department patients with and without in-hospital cardiac arrest (IHCA) within one hour

Variables	Total (N = 832,226)	No IHCA (N = 831,353)	IHCA (N = 873)	p-value
Age	45.0 (32.0 – 59.0)	45.0 (32.0 – 59.0)	63.0 (53.0 – 75.0)	< 0.01
Male	404,230 (48.6)	403,591 (48.5)	639 (73.2)	< 0.01
MAP (mmHg)†	93.3 (86.7 – 103.7)	93.3 (86.7 – 103.7)	90.7 (73.0 – 106.7)	< 0.01
Pulse rate, bpm†	80.0 (72.0 – 88.0)	80.0 (72.0 – 88.0)	92.0 (76.0 – 110.0)	< 0.01
Respiratory rate, breaths per min†	20.0 (18.0 – 20.0)	20.0 (18.0 – 20.0)	20.0 (18.0 – 22.0)	< 0.01
LOS in ED (hours)	0.5 (0.2 – 0.8)	0.5 (0.2 – 0.8)	0.7 (0.6 – 0.9)	< 0.01
Cancer	23,071 (2.8)	23,035 (2.8)	36 (4.1)	< 0.01
Not alert††	25,995 (3.1)	25,458 (3.1)	537 (61.5)	< 0.01
KTAS level 1	18,816 (2.3)	18,274 (2.2)	542 (62.1)	< 0.01
ICU admission	17,927 (2.2)	17,289 (2.1)	638 (73.1)	< 0.01
Mortality	3,346 (0.4)	3,136 (0.4)	210 (24.1)	< 0.01

Data are presented as median (interquartile range [IQR]) or as number (percentage). † Vital signs were checked on ED arrival. †† Glosgow Coma Scale < 13. Abbreviations: IHCA = in-hospital cardiac arrest; MAP = mean arterial pressure; ED = emergency department; KTAS = Korean triage acuity scale; ICU = intensive care unit.

Table S2. Baseline demographic and clinical characteristics of emergency department patients with in-hospital cardiac arrest (IHCA) within and after one hour

Variables	After 1 hour (N = 9,180)	Within 1 hour (N = 873)	p-value
Age	68.0 (55.0–78.0)	63.0 (53.0 – 75.0)	< 0.01
Male	6,036 (65.8)	639 (73.2)	< 0.01
MAP (mmHg)†	87.3 (70.0–104.0)	90.7 (73.0 – 106.7)	< 0.01
Pulse rate, bpm†	95.0 (78.0–114.0)	92.0 (76.0 – 110.0)	< 0.01
Respiratory rate, breaths per min†	20.0 (18.0–22.0)	20.0 (18.0 – 22.0)	< 0.01
LOS in ED (hours)	4.2 (2.4–9.3)	0.7 (0.6 – 0.9)	< 0.01
Cancer	1,032 (11.2)	36 (4.1)	< 0.01
Not alert††	5,006 (54.5)	537 (61.5)	< 0.01
KTAS level 1	4,416 (48.1)	542 (62.1)	< 0.01
ICU admission	5,375 (58.6)	638 (73.1)	< 0.01
Mortality	3,277 (35.7)	210 (24.1)	< 0.01

Data are presented as median (interquartile range [IQR]) or as number (percentage). † Vital signs were checked on ED arrival. †† Glosgow Coma Scale < 13. Abbreviations: IHCA = in-hospital cardiac arrest; MAP = mean arterial pressure; ED = emergency department; KTAS = Korean triage acuity scale; ICU = intensive care unit.

Table S3. Top 10 categories for ED diagnosis

No IHCA (N = 5,817,436)		IHCA (N = 9,180)	
**Diagnosis**	**Frequency**	**Diagnosis**	**Frequency**
Gastrointestinal	890,185 (15.3)	Primary cardiac (non-AMI)	2,383 (26.0)
Nontraumatic neurologic disorder	471,379 (8.1)	Primary respiratory	1,629 (17.7)
Primary respiratory	219,586 (3.8)	AMI	1,377 (4.1)
Urology	171,588 (2.9)	Others	154 (1.7)
Unspecified fever	108,365 (1.9)	Sepsis	153 (1.7)
Others	94,885 (1.6)	Gastrointestinal	142 (1.3)
Urticaria	77,566 (1.3)	Cerebrovascular	134 (1.4)
Dyspnea	51,611 (0.9)	Chronic kidney disease	75 (0.1)
Primary cardiac	49,089 (0.1)	Trauma	61 (0.1)
Trauma	46,565 (0.1)	Acute Kidney Injury	58 (0.1)

Data are presented as number (percentage). Abbreviations: ED = emergency department; IHCA = in-hospital cardiac arrest; AMI = acute myocardial infarction.
